# Clinical performance validation of *PITX2* DNA methylation as prognostic biomarker in patients with head and neck squamous cell carcinoma

**DOI:** 10.1371/journal.pone.0179412

**Published:** 2017-06-15

**Authors:** Verena Sailer, Heidrun Gevensleben, Joern Dietrich, Diane Goltz, Glen Kristiansen, Friedrich Bootz, Dimo Dietrich

**Affiliations:** 1 Department of Pathology and Laboratory Medicine, Weill Cornell Medicine, New York, New York, United States of America; 2 Caryl and Israel Englander Institute for Precision Medicine, Weill Cornell Medicine, New York, New York, United States of America; 3 Institute of Pathology, University Hospital of Bonn, Bonn, Germany; 4 Department of Otolaryngology, Head and Neck Surgery, University Hospital Bonn, Bonn, Germany; 5 Institute of Pathology, University Hospital of Cologne, Cologne, Germany; University of North Carolina at Chapel Hill School of Medicine, UNITED STATES

## Abstract

**Background:**

Despite advances in combined modality therapy, outcomes in head and neck squamous cell cancer (HNSCC) remain dismal with five-year overall survival rates of less than 50%. Prognostic biomarkers are urgently needed to identify patients with a high risk of death after initial curative treatment. Methylation status of the paired-like homeodomain transcription factor 2 *(PITX2)* has recently emerged as a powerful prognostic biomarker in various cancers. In the present study, the clinical performance of *PITX2* methylation was validated in a HNSCC cohort by means of an independent analytical platform (Infinium HumanMethylation450 BeadChip, Illumina, Inc.).

**Methods:**

A total of 528 HNSCC patients from The Cancer Genome Atlas (TCGA) were included in the study. Death was defined as primary endpoint. *PITX2* methylation was correlated with overall survival and clinicopathological parameters.

**Results:**

*PITX2* methylation was significantly associated with sex, tumor site, p16 status, and grade. In univariate Cox proportional hazards analysis, *PITX2* hypermethylation analyzed as continuous and dichotomized variable was significantly associated with prolonged overall survival of HNSCC patients (continuous: hazard ratio (HR) = 0.19 [95%CI: 0.04–0.88], p = 0.034; dichotomized: HR = 0.52 [95%CI: 0.33–0.84], p = 0.007). In multivariate Cox analysis including established clinicopathological parameters, *PITX2* promoter methylation was confirmed as prognostic factor (HR = 0.28 [95%CI: 0.09–0.84], p = 0.023).

**Conclusion:**

Using an independent analytical platform, *PITX2* methylation was validated as a prognostic biomarker in HNSCC patients, identifying patients that potentially benefit from intensified surveillance and/or administration of adjuvant/neodjuvant treatment, i.e. immunotherapy.

## Introduction

With an annual incidence of approximately 61,760 cases and 13,190 estimated deaths, head and neck squamous cell carcinoma (HNSCC) is ranked among the leading causes of cancer-related death in the United States [1]. While tobacco and alcohol abuse traditionally are the primary risk factors for HNSCC, a subset of tumors with oropharyngeal location is strongly associated with high risk human papilloma virus (HPV) infections [2]. HPV-positive (HPV+) cancers are more responsive to chemotherapy and radiation, and patients have shown improved overall survival rates compared to HPV-negative (HPV-) cancer patients [3]. The underlying molecular mechanisms for this clinical observation, however, are not fully understood. In a first comprehensive study of genomic alterations in 279 HNSCC patients, smoking-related tumors frequently harbored loss of function mutations of *TP53* and *CDKN2A* in addition to multiple copy number alterations, whereas HPV-associated tumors were predominantly characterized by activating *PIK3CA* mutations [4]. Mechanisms explaining the differing treatment response of HPV+ and HPV- HNSCC, however, still need to be elucidated, particularly in the light of emerging alternative HNSCC treatment concepts.

Despite initial treatment with curative intent, recurrence rates in HNSCC patients remain high leading to local or distant recurrences in 30% and 25%, respectively, and five-year overall survival rates of less than 50% [5–6]. Reliable prognostic biomarkers are urgently needed to identify patients at risk of disease recurrence and subsequent death, as these patients might beefit from an intensified first-line treatment and surveillance. Current treatment regimens, including surgery, definite or adjuvant radiochemotherapy, elicit a number of severe side effects leaving little room for treatment intensification [7–8]. Emerging therapeutic strategies, e.g. anti-PD-1 antibodies pembrolizumab and nivolumab, which have recently been approved for the treatment of advanced and metastatic HNSCC, are promising options for the management of high risk patients [9–10]. In 2006, the anti-EGFR monoclonal antibody cetuximab was further approved for the treatment of localized disease in combination with radiotherapy and as a single agent in metastatic and recurrent cancers [11]. Particularly for high-risk patients, employing cetuximab in an adjuvant setting has been shown to be feasible [12]. Identifying high-risk patients, however, remains a challenge for both the surgical pathologist and the clinician. So far, only few prognostic biomarkers have shown promising potential. The detection of HPV in tumor tissue, for instance, has been reported as a strong prognostic biomarker predicting reduced risk of death [13]. In a study of 141 HNSCC patient samples, truncating mutations of *TP53* resulting in loss of function of the tumor suppressor gene were further associated with worse overall survival (HR = 2.54, p = 0.008) [14]. The investigation of DNA promoter methylation to identify biomarkers in formalin-fixed paraffin-embedded (FFPE) tissue has shown encouraging results. In brief, epigenetic changes are mediated through binding of a methyl- or hydroxymethyl-group to a cytosine-phosphate-guanin (CpG)-dinucleotide. Clustered CpG-dinucleotides, CpG-islands, are found in promoter regions and their methylation or demethylation modulates gene activity [15].

Previously, the methylation status of the paired-like homeobox transcription factor 2 (*PITX2)* and its adjacent long non-coding RNA (*PANCR*) have been described as promising tissue-based biomarkers in HNSCC, and *PITX2* hypermethylation has been associated with better overall survival in HNSCC patients [16]. Mutation of the *PITX2 gene*, located on chromosome 4q25, causes the developmental disorder Axenfeld-Rieger syndrome type I, which is characterized by ophtalmological and cardiovascular abnormalities and dental hypoplasia [17–18]. In recent years, *PITX2* has attracted interest as a potential biomarker in malignant tumors, i.e. non-small cell lung cancer (NSCLC), biliary tract cancers, prostate cancer, and breast cancer [16, 19–27]. In NSCLC, *PITX2* hypermethylation was identified as significant predictor of progression-free survival [28]. In hormone receptor-positive, lymph node-negative breast cancer, high levels of *PITX2* methylation were, in contrast, associated with a high risk of recurrence [25–27]. In addition, *PITX2* methylation has been shown to be predictive of response to adjuvant anthracycline-based chemotherapy in node-positive, hormone receptor-positive breast cancer patients [24]. Therawis GmbH (Munich, Germany) in collaboration with Qiagen N.V. (Venlo, The Netherlands) have recently announced the development of a commercially available assay exploiting the additional predictive potential of *PITX2* methylation in breast cancer patients. In prostate cancer, *PITX2* hypermethylation has been associated with biochemical recurrence, contributing to individualized risk assessment as a single assay and in combination with *PITX3* methylation analysis [20, 22–23, 29]. In addition to its known role in the development of several organs, *PITX2* also mediates cell cycle progression by regulating the transcription of cyclin A1, cyclin D2, and p21, therefore providing sufficient evidence to implicate *PITX2* in tumorigenesis [30–32]. Moreover, *PITX2* is over-expressed on a protein level in a number of solid tumors and has been associated with cancer progression [33]. Several studies have demonstrated that *PITX2* methylation can be quantified accurately and robustly in various clinically relevant specimens, i.e. FFPE biopsies, FFPE sections, and microdissected cells from FFPE sections [28, 34–37]. Hence, *PITX2* methylation is an ideal candidate for the implementation into a clinical routine setting.

The Cancer Genome Atlas (TCGA) has provided an unprecedented platform for researchers to study genomic, transcriptomic, proteomic, and methylomic data across a multitude of different cancer types [38]. Given the need for biomarkers for treatment stratification in HNSCC patients and the encouraging results regarding *PITX2*, this study aimed at validating the clinical performance of *PITX2* methylation for the outcome prediction of HNSCC patients by means of an independent analytical platform, i.e. Infinium HumanMethylation450 BeadChip, in the TCGA HNSCC patient cohort.

## Materials and methods

### Ethical approval

The present study is based entirely upon data generated by the TCGA research network (www.cancergenome.nih.gov). All patients included in TCGA have been enrolled following strict human subjects protection guidelines, informed consent and IRB (Institutional Review Board) review of protocols. All patients provided informed consent (written).

### Patients

All head and neck squamous cell carcinoma patients (n = 528) from the TCGA cohort (Project Id: TCGA-HNSC) were included into the present study (https://portal.gdc.cancer.gov/projects/TCGA-HNSC). Survival was defined as time to death by any cause (overall survival, OS) and censored after five years (1,825 days).

### Statistical analyses

Gene methylation data were publicly available and downloaded from the UCSC Xena browser (www.xena.ucsc.edu). Methylation analysis was performed using the Infinium HumanMethylation450 BeadChip Kit (Illumina, Inc., San Diego, CA, USA). Methylation levels were calculated as previously described [39–41]. For statistical analysis, SPSS version 24 (SPSS Inc., Chicago, IL, USA) was used. Survival analyses were conducted by Kaplan-Meier and Cox proportional hazard regression analyses. P-values refer to Wald and log-rank tests, respectively. The correlation between age and methylation was tested using the Spearman’s rank correlation coefficient (ρ). For comparison between groups, Mann-Whitney U test, one-way analysis of variance (one-way ANOVA), and Fisher’s exact test were employed. P-values lower than 0.05 were considered significant.

## Results

### *PITX2* promoter methylation in HNSCC patients

Gene methylation data from 528 patients with histologically confirmed HNSCC were available, including tumor tissues in all cases and matched normal adjacent tissues in 50 cases (9.5%). Histological tumor subtypes comprised 517 (97.9%) squamous cell cancers of the usual type, ten (1.9%) cases of the basaloid type, and one (0.2%) spindle cell variant.

**Fig 1:**
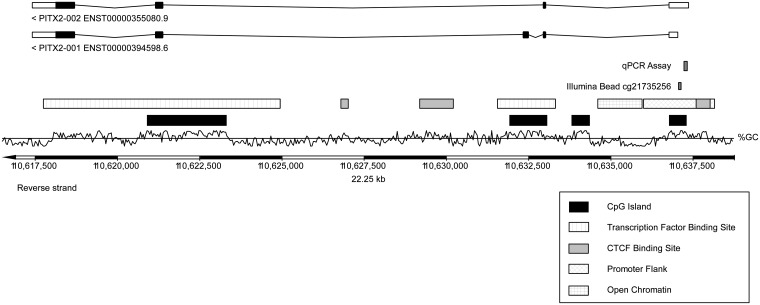
Organization of the *PITX2* gene. Genomic organization of the *PITX2* gene and locations of the *PITX2* qPCR assay [16] and the Illumina HumanMethylation450 BeadChip bead cg21735256. The information was taken from Ensembl Homo sapiens version GRCh38.p7.

A previous report employing quantitative real-time PCR (qPCR) assays investigated four CpG-dinucleotides within the sequence context CGGGAGCCGGAGCCGGGAGAGCG located on chromosome 4:110637267–110637289 (Ensembl.org genome assembly: GRCh38.p10) [16]. In the current TCGA dataset, the Illumina HumanMethylation450 BeadChip bead cg21735256 was analyzed. This bead is located in the vicinity (approximately 200 base pairs distance) of the previously published CpG-dinucleotides and belongs to the same CpG-island (Fig 1). Accordingly, methylation of the cg21735256 bead was assumed to have the same transcriptional consequences for the *PITX2* gene. *PITX2* DNA methylation ranged from 2.1% to 67.5% (mean: 13.0%, median: 8.2%) in tumor tissue and from 4.5% to 14.4% (mean: 8%, median: 7.5%) in normal tissue. A significant difference in methylation levels was detected when comparing tumor and normal tissue (p = 0.003).

### Association of *PITX2* promoter methylation with clinicopathological parameters

Significant differences in *PITX2* methylation status were found between male and female patients in the TCGA HNSCC cohort (p = 0.026). This may in part be explained by a difference in smoking habits with female patients being mainly non-smokers (Fisher’s exact test, p < 0.001). No difference in pack years was seen in the patients who reported a history of smoking (p = 0.35). Further, no gender difference was observed regarding HPV-status (p = 0.15). However, HPV-status was only reported for a fifth (19.9%) of all samples, so this result may well be skewed. Nonetheless, there was a difference in *PITX2* DNA methylation in HPV- versus HPV+ patient samples with the latter patient group showing significantly higher methylation levels (p < 0.001). This finding was in concordance with previously reported data [16]. Additionally, HPV+ tumors were more frequently located in the oropharynx, and oroparyngeal cancers presented with significantly higher methylation levels compared to tumors from other sites (p < 0.001) [42]. Poorly differentiated and undifferentiated cancers had significantly higher methylation levels compared to well or moderately differentiated tumors (p < 0.001). No difference in *PITX2* methylation was found for age, smoking status, and number of pack years (analyzing males and females together), history of alcohol consumption, tumor (T) and nodal (N) categories, race, and surgical margin status (Table 1).

**Table 1:** Association of *PITX2* methylation with clinicopathological characteristics. Association of *PITX2* methylation with clinicopathological characteristics in HNSCC patients of the TCGA cohort (n = 528).

Characteristic		No. [%] of patients	Mean *PITX2* methylation [mean ± standard deviation)	*p*-value
**All patients**		528 (100)	12.7 (±10.9)	
**Sex**				0.026*
	Female	142 (26.9)	10.9 (±8.1)	
	Male	386 (73.1)	13.3 (±11.7)	
**Age (years)**				0.65
	Mean	61		
	Median	61		
	n ≤ Median	282 (53.4)	13.0 (±11.6)	
	n > Median	245 (46.4)	12.3 (±10.2)	
	Unknown	1 (0.2)		
**Smoking status**				0.61
	Non-Smoker	122 (23.1)	13.2 (±11.6)	
	Smoker	393 (74.4)	12.6 (±10.8)	
	Unknown	13 (2.5)		
	Pack years			0.18
	(≤ 40)	168 (31.8)	13.5 (±11.5)	
	(> 40)	130 (24.6)	11.7 (±10.2)	
	Unknown	230 (43.6)		
**History of alcohol consumption**				0.99
	Yes	352 (66.7)	12.7 (±11)	
	No	165 (31.3)	12.7 (±11)	
	Unknown	11 (2.1)		
**Tumor site**				<0.001*
	Oral cavity	250 (47.3)	9.9 (±7.6)	
	Oropharynx	151 (28.6)	16.5 (±14.4)	
	Hypopharynx	10 (1.9)	14.4 (±6.6)	
	Larynx	177 (22.2)	13.7 (±9.5)	
**Race**				0.66
	White	452 (85.6)	13 (±11.3)	
	Black or African American	48 (9.1)	10.9 (±7.9)	
	Asian	11 (2.1)	11.7 (±9.3)	
	American Indian or Alaska Native	2 (0.4)	17.8 (±13)	
	Unknown	15 (2.78)		
**Pathologic tumor category (pT)**				0.15
	Tis/T1/T2	190 (36)	12.7 (±10.4)	
	T3/T4	276 (52.3)	11.3 (±9.8)	
	Unknown	62 (11.7)		
**Pathologic nodal category (pN)**				0.42
	N0	180 (34.1)	12.1 (±9.7)	
	N1	68 (12.9)	10.8 (±8)	
	N2	172 (32.6)	11.0 (±9.5)	
	Unknown	108 (20.5)		
**p16**				<0.001*
	Negative	74 (14)	9.7 (±6.5)	
	Positive	41 (7.8)	25.7 (±16.2)	
	Unknown	413 (78.2)		
**Grade (G)**				<0.001*
	G1	63 (11.9)	9.8 (±6.3)	
	G2	311 (58.9)	11.3 (±9.2)	
	G3	125 (23.7)	14.9 (±13.3)	
	G4	7 (1.3)	28.5 (±11.1)	
	Unknown	22 (4.2)		
				0.003*
	G1 and G2	374 (70.8)	11.1 (±8.8)	
	G3/G4	132 (25)	15.6 (±13.5)	
**Surgical margin**				0.83
	Negative	407 (77.1)	11.7 (±9.8)	
	Positive	60 (11.4)	12.0 (±11.1)	
	Unknown	61 (11.6)		

Mann-Whitney U test for sex, smoking status, history of alcohol consumption, pT, p16, grade (dichotomized), surgical margin; One-Way ANOVA for tumor site, pN, grade, race; Spearman’s rank correlation for age, pack years.

* significant feature

### *PITX2* methylation status as independent prognostic factor for overall survival

**Fig 2:**
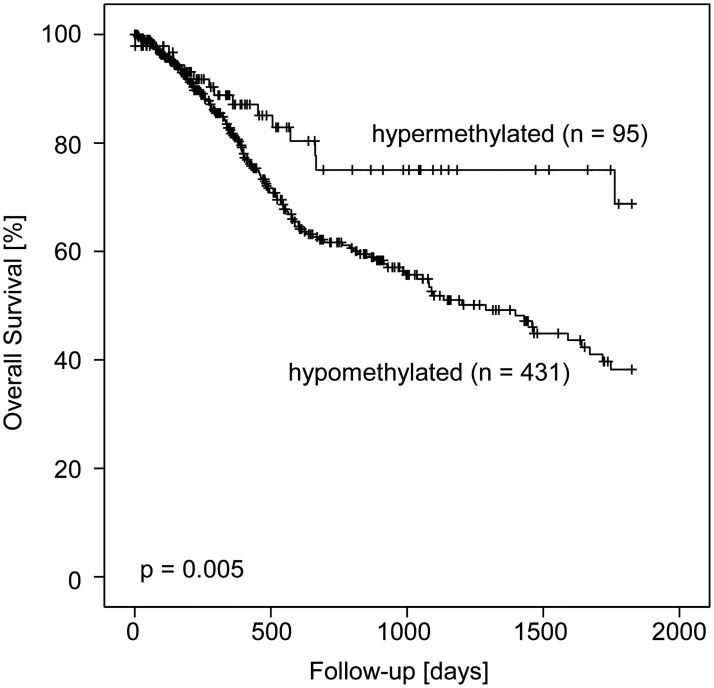
Kaplan-Meier survival analysis. Kaplan-Meier survival analysis of overall survival in 528 HNSCC patients stratified according to *PITX2* DNA methylation status. Overall survival in patients with *PITX2* hypermethylated HNSCC was significantly improved compared to patients with *PITX2* hypomethylated tumors.

In order to avoid overfitted results, Cox regression analysis was performed using *PITX2* methylation as a continuous variable. Univariate analysis revealed a significantly reduced risk of death for patients with hypermethylated tumors (hazard ratio (HR) = 0.19 [95%CI: 0.04–0.88], p = 0.034). For the dichotomization of DNA methylation values, patients were stratified according to an optimized cut-off (20.3%) using a publicly available cut-off calculator [43]. As a result, 433 (82.0%) tumor samples were assigned to the *PITX2* hypomethylated group, and 95 (18.0%) specimens were allocated to the *PITX2* hypermethylated group. In univariate Cox proportional hazards analysis, patients with hypermethylated tumors had a significantly reduced risk of death compared to patients with hypomethylated tumors (HR = 0.52 [95%CI: 0.33–0.84], p = 0.007). These findings were further confirmed in Kaplan-Meier survival analysis (p = 0.005, Fig 2). In multivariate analysis, *PITX2* methylation added significant prognostic information to established clinicopathological parameters, i.e. age, T and N category (Table 2). Although poorly differentiated tumors had significantly higher methylation levels in our study, histological tumor grading did not add further independent information in survival analyses.

**Table 2:** Univariate and multivariate Cox proportional hazards analyses. Univariate and multivariate Cox proportional hazards analyses on overall survival in 528 HNSCC patients. Multivariate Cox proportional hazard analysis was conducted including only variables that showed significance in univariate analysis (*PITX2* methylation [dichotomized variable; cut off: 20.3%], T category, N category, age).

Variable	Univariate Cox		Multivariate Cox	
	HR (95% CI)	*P*-value	HR (95% CI)	*P*-value
*PITX2* methylation (continuous)	0.19 (0.04–0.88)	0.034*	NA	NA
*PITX2* methylation (dichotomized)	0.52 (0.33–0.84)	0.007*	0.28 (0.09–0.84)	0.023*
pT3/4 vs. pT1/2	1.57 (1.14–2.17)	0.004*	1.84 (1.20–2.84)	0.006*
pN1/2 vs. pN0	1.62 (1.12–2.35)	0.010*	1.50 (1.01–2.20)	0.044*
Age (continuous variable)	1.02 (1.01–1.03)	0.003*	1.02 (1.00–1.04)	0.044*
p16 (positive vs. negative)	0.66 (0.18–2.48)	0.54	NA	NA
Grade (G3,G4 vs. G1,G2)	0.87 (0.70–1.07)	0.18	NA	NA
Surgical margin (positive vs. negative)	1.45 (0.97–2.16)	0.17	NA	NA

NA: Not applicable, variate not included into multivariate analysis

* significant feature

## Discussion

HNSCC is a common cancer type with a dismal prognosis in the event of recurrent or metastatic disease. Consequently, there is a pressing need for reliable prognostic biomarkers which might aid the risk stratification of patients and clinical decision-making process with regard to primary treatment and subsequent surveillance. Methylation analysis of *PITX2* has recently been shown to predict overall survival in HNSCC patients [16]. Encouraged by these data, we sought to investigate *PITX2* methylation in an independent HNSCC cohort by means of an additional innovative technology.

In the present study, *PITX2* DNA hypermethylation was associated with improved overall survival, thereby validating our previous results in the TCGA dataset. Also in line with prior results, *PITX2* methylation further added independent prognostic information to established clinicopathological parameters like age, T and N category. Combining both studies, there is now strong evidence for the high analytical performance and prognostic value of *PITX2* methylation in almost one thousand HNSCC patients. Implementing *PITX2* methylation into prospective clinical trials is therefore highly desirable to further support these findings. The robust prognostic performance of *PITX2* as biomarker in HNSCC is further corroborated, since different CpG-sites were analyzed in the present study, hereby demonstrating that the prognostic value is not only limited to the previously published CpG-sites [16]. In earlier publications, *PITX2* testing was conducted using qPCR and DNA microarray methodologies [34–35]. The employment of methylation data generated by means of the Illumina HumanMethylation450 BeadChip poses an additional strength of the present study, as the application of different diagnostic platforms not only increases the value of a biomarker but also allows for an area-wide implementation of the biomarker into clinical routine.

The biological function of *PITX2* in tumorigenesis has been linked to its role as key player in cell cycle regulation. Hypermethylation is assumed to result in silencing of the *PITX2* gene, therefore disrupting cell cycle progression and proliferation. The same phenomenon has been shown for NSCLC [28], a tumor which, like HNSCC, is known for its strong association with a history of smoking. Interestingly, in hormonally dependent tumors like breast and prostate cancer, *PITX2* DNA hypermethylation has been associated with adverse overall survival [25–27, 29]. This might point at a role of *PITX2* in tumorigenesis which lies beyond cell cycle regulation. However, it remains unclear how methylation actually affects gene transcription. Wang and colleagues could not identify a correlation between low *PITX2* protein expression and methylation in pancreatic ductal adenocarcinoma but rather suggested that *PITX2* acts as a tumor suppressor by inhibiting the TGFβ1-Smad4-pathway [44]. In embryonic development, isoforms of *PITX2* exert various functions in different organs, thus possibly providing an explanation for its diverse and sometimes opposing role in tumorigenesis [45].

In HNSCC, *PITX2* DNA-methylation is significantly associated with HPV status and tumor site. This underscores that HPV-associated tumors are molecularly distinct from smoking-associated tumors. It has also been established by now that integration of HPV into the genome might result in altered DNA methylation [46–47]. Thus, it cannot be ruled out that *PITX2* DNA methylation acts as a surrogate marker for HPV infection. Neither HPV status nor the tumor site was associated with survival in the present study. Information on HPV status, however, was missing for the majority of the cohort. Thus, further studies are needed to investigate the association of *PITX2* methylation and HPV.

Three monoclonal antibodies targeting either EGFR (cetuximab) or PD-1 (pembrolizumab, nivolumab) are currently available for the treatment of HNSCC patients. Only very recently, the immune checkpoint inhibitors pembrolizumab and nivolumab have been approved for patients in a recurrent or metastatic disease setting. However, they might potentially be a therapeutic option for high-risk patients with tumors harboring *PITX2* hypomethylation as part of a neoadjuvant or adjuvant therapeutic regimen. With regard to the findings in the present study, evidence is mounting that *PITX2* DNA methylation might serve as highly informative prognostic biomarker in HNSCC patients. A prospective randomized trial stratifying patients according to classical and molecular risk factors, however, would need to confirm the predictive value of *PITX2* DNA methylation status in HNSCC patients.
